# Predicting suitable habitat of swamp deer (*Rucervus duvaucelii*) across the Western Terai Arc Landscape of Nepal

**DOI:** 10.1016/j.heliyon.2023.e16639

**Published:** 2023-05-25

**Authors:** Bijaya Dhami, Binaya Adhikari, Saroj Panthi, Bijaya Neupane

**Affiliations:** a Tribhuvan University, Institute of Forestry, Pokhara, Kaski, 33700, Nepal; b Pokhara Zoological Park and Wildlife Rescue Center, Kaski, 33700, Nepal; c Ministry of Forest, Environment and Soil Conservation, Gandaki, 33700, Nepal

**Keywords:** Swamp deer, MaxEnt, Habitat, TAL, Conservation

## Abstract

Over the last few years, intensifying human impact and the deterioration of natural habitats have severely restricted the global distribution of large herbivores. *Rucervus duvaucelii,* commonly recognized as the swamp deer, is a habitat-specialist endemic large herbivore of the Indian Subcontinent. It is classified as vulnerable by the IUCN and listed in CITES Appendix I due to a steep decline in its population, which is primarily due to anthropogenic causes. In Nepal, the last remaining population of this species is confined to limited pocket areas within the western Terai Arc Landscape. We explored potential habitat for swamp deer across this landscape using species distribution modelling through the MaxEnt algorithm by using 173 field-verified presence points alongside six anthropogenic, four topographic, and four vegetation-related variables. Our study found that out of the total study area (9207 km^2^), only 6% (590 km^2^) was suitable for swamp deer. Approximately 45% of suitable habitat was incorporated within protected areas, with Shuklaphanta National Park harboring the largest habitat patch. The suitability of habitat was discovered to be positively associated with low-elevation areas, areas near water sources, and areas far from settlements, implying the need to conserve water sources and minimize the extension of anthropogenic pressure for their long-term conservation. Additionally, we suggest the implications of a swamp deer-centric conservation strategy, with an emphasis on increasing connectivity through the corridors and landscape-level population connectivity through *trans*-boundary conservation initiatives between Nepal and India. Moreover, considering large herbivores' high vulnerability to extinction, similar researche incorporating anthropogenic factors is of the utmost importance to produce vital information on habitat suitability for conserving other regionally and globally endemic, habitat-specialized herbivores.

## 1 Introduction

Large herbivores exhibit a significant role in molding the ecosystem through ecological function that benefits other species [1,2]. Despite their critical role in the ecosystem, various anthropogenic pressures such as habitat degradation, resource exhaustion, poaching, and human-wildlife conflicts [[3], [4], [5], [6], [7], [8]] have posed a threat of extinction to 60% of the world's large herbivores [8,9]. Although the South Asia holds preeminent varieties of terrestrial mega herbivores [10,11], 12 out of 15 terrestrial large herbivores are confronting critical conservation hurdles, as they are delegated “threatened” by the IUCN [2,8], owing to severe loss of their habitat and significant population decline.

Swamp deer (*Rucervus duvaucelii*), commonly referred as Barasingha, are habitat-specialized large grassland herbivores of the order Artiodactyla and the family Cervidae [12]. *R. duvaucelii* is primarily a grazer [13] but occasionally feeds on aquatic plants [14]. It heavily prefers short grassland [15] and principally feeds on *Saccharum* spp., *Narenga porphyrocoma*, *Cynodon dactylon*, *Imperata cylindrica*, *Oryza rufpogon, and Phragmites karka* [13,14,16,17]. *R. duvaucelii* prefers grasslands with water holes [15]. This preference is due to their requirement for water, as they engage in drinking activities at least twice daily during the winter and monsoon seasons, and three or more times during the summer season [17]. *R. duvaucelii* is usually known to move 2–3 km a day [18,19], but is acknowledged to avoid thickly forested areas [13]. The IUCN Red List of threatened species designates the species as globally vulnerable [12] and categorized as endangered on the National Red List of mammals at the national level [20,21]. It is also listed in Appendix I by CITES and protected under the National Parks and Wildlife Conservation Act 1973 of Nepal [22]. recognized three subspecies of *R. duvaucelii,* namely, *Rucervus duvaucelii duvaucelii, Rucervus duvaucelii branderi*, and *Rucervus duvaucelii ranjitsinhi*. *R. d. duvaucelii* (hereafter swamp deer) is the most abundant subspecies, contributing to around 80% of the worldwide population [19,23].

The distribution range of swamp deer encompasses the northern region of the Indo-Gangetic plain, including Nepal and various parts of India along the Ganges [24]. Generally, undisturbed patches of grassland are reported to encompass a substantial number of the swamp deer [12,25]. The swampy grassland within flood plains of Nepal contains the most extensive and suitable habitat for swamp deer [26,27]. In Nepal, the sole surviving population of swamp deer is now restricted to the pocket areas of Bardia National Park (BNP) and Shuklaphanta National Park (SNP), with an estimate of 2325 individuals [21]. With the majority of the population contained within the Terai Arc Landscape (TAL), SNP is thought to have the world's largest swamp deer herd [28,29], making the area critical for conservation [25,30].

In Nepal, swamp deer research has so far focused on a variety of topics, including abundance and density [[31], [32], [33], [34]], the impact of construction [35], activity pattern [17], habitat use [13,15,36], habitat suitability [24]. However, there is a dearth of insights regarding the present distribution status of swamp deer in Nepal. Moreover, the information on distribution of swamp deer in human-dominated landscapes and their response to anthropogenic variables are sparsely known. Since swamp deer is a habitat specialist endemic species [36,37], information regarding its potential distribution, taking anthropogenic aspects into consideration, can be deemed crucial for prioritizing conservation actions.

Escalating anthropogenic activities such as poaching for trophies and food assumption [[38], [39], [40], [41]], deforestation and forest fire [42,43], change in river dynamics, increase in siltation [44], habitat fragmentation [45], infrastructure development [46], disease [29,36,40,41], human-wildlife conflict [47,48], and food deficiency due to agricultural expansion [49] have become a major threat to biodiversity conservation globally. These activities are especially likely to endanger the existence of habitat specialist species like swamp deer, which are already threatened with extinction [50]. Thus, knowledge of human triggered and ecological drivers that influence the habitat use and distribution of threatened wildlife species is crucial for habitat management and conservation [[51], [52], [53], [54], [55], [56]]. These ecological drivers include topography and micro-climate (elevation, slope, aspect, and water distance) and anthropogenic-related factors (distance to human paths, roads, settlements, land use, and population density). These factors could potentially influence the long-term habitat composition, structure, or function, that can be identified with the help of species distribution modeling [52,[57], [58], [59], [60]], as well as potentially weaken species resilience to future disturbances [[61], [62], [63]].

With the evolution of 3S techniques (GIS, GPS, and RS), numerous models have been launched to document the distribution of suitable habitat. These models encompass mechanism models [64,65], regression models [66], and ecological niche models [66,67]. Recognized for its effectiveness in predicting wildlife habitat distribution and risk zone modeling [[68], [69], [70], [71]], the maximum entropy model (MaxEnt) has emerged as an acclaimed ecological niche model. Originally, the MaxEnt model was developed to quantify the presence density of specific species across the landscape [67], but over the years it has been used to predict species distribution pattern and ecological niches, utilizing presence-only data and environmental predictors, thus minimizing bias and elevating accuracy [67,72,73]. Some literature has discussed a few limitations of MaxEnt, including potential biases in the sampling of occurrence points, the complexity of feature selection, and the regularization multiplier [74]. Nevertheless, in terms of performance, this model is generally considered superior to others [75].

This study aimed to investigate the habitat suitability of vulnerable swamp deer using MaxEnt by assessing the contribution of ecological and anthropogenic variables with respect to their present distribution. Since small, isolated populations in patches are far more prone to becoming extinct as a result of demographic and environmental stochasticity, or random events of chance [76,77], it is a dire necessity to identify the potential suitable habitats of endemic large herbivores to augment those favorable characteristics and improve connectivity among habitats for sustainable conservation through the collaborative efforts of government and local people. Therefore, our study aimed to identify the existing distribution as well as viable habitats for new populations of swamp deer using a well-known species distribution model by incorporating anthropogenic, topographic, and vegetation-related variables.

## 2 Materials and method

### 2.1 Study area

**Fig. 1:**
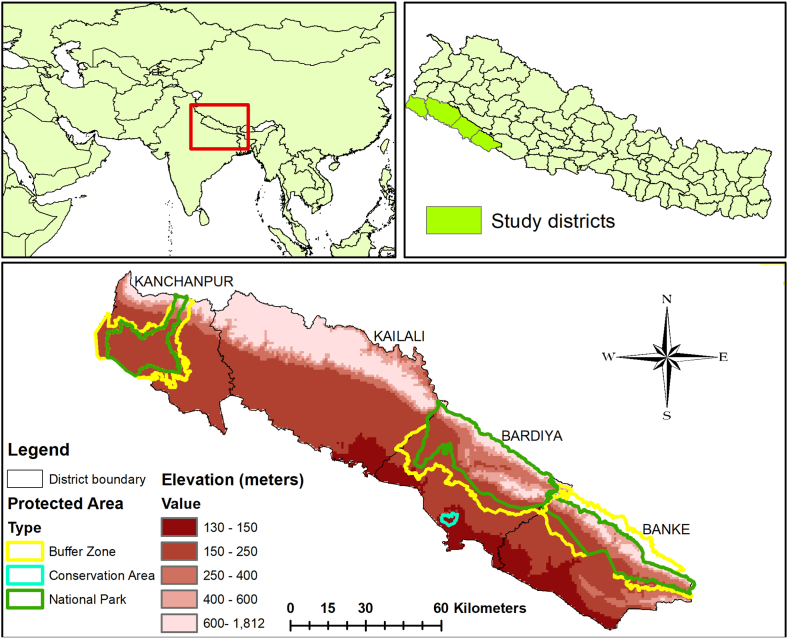
Map depicting study area with elevation gradient and type of protected areas within four districts of western TAL (Terai Arc Landscape).

The study was carried out within the western segment of Nepal's Terai Arc Landscape, including Banke, Bardia, Kailali, and Kanchanpur Districts, with an area of 9207 km^2^ (Fig. 1). SNP and its buffer zone (BZ), BNP and its BZ, Banke National Park (BaNP) and its BZ, and Krishnasar Conservation Area (KrCA) are the protected areas within the study area [78]. Around 30% (2754 km^2^) of the study area is encompassed by protected areas [28]. According to the IUCN's Protected Area Categories System, national parks go into category II, whereas conservation areas and buffer zones fall into category VI (www.iucn.org). In national parks, admission without authorization from the park management is prohibited. However, reasonable entry is allowed for locals to generate their subsistence needs in conservation areas and its buffer zone. The lowlands of Nepal are rich in floral and faunal diversity and have noteworthy conservation value [4,79,80]. Sal (*Shorea robusta*), Asna (*Terminalia tomentosa*), Botdhamero (*Lagestroemia parviflora*), and Sindure (*Mallatus Philippines*) are the dominant floral species [81]. Similarly, Swamp deer (*Cervus duvaucelii*), Black buck (*Antilope cervicapra*), hog deer (*Axis porcinus*), Python (*Python molurus*), Spotted deer (*Axis axis*), Sambar deer (*Rusa unicolor*), Gaur (*Bos gaurus*), Rhinoceros (*Rhinoceros unicornis*), Tiger (*Panthera tigris tigris*), Common leopard (*Panthera pardus*), Wild Asian elephant (*Elephas maximas*), and Giant hornbill (*Buceros bicornis*) are the major faunal species [28,36,48].

## 3 Data collection

### 3.1 Swamp deer presence points

The data on presence points of swamp deer were collected during the period from 3rd February to 7^th^ April 2021. We held discussions with local people, officials of division forest offices, and officials of protected areas to identify potential habitats for swamp deer and then visited those areas to record the evidence of the presence of swamp deer. Direct observation of individuals, pellet droppings, hoofmarks, and the presence of carcasses were the major methods of determining the presence of the species [82]. Swamp deer were identified with the help of their distinct antlers. Unlike other species, in the top part of the antler beam of swamp deer, the branches are arranged in a dichotomous way. Similarly, their hoof prints have a characteristic splayed pattern, showcasing their adaptive trait for thriving in marshy habitats. While swamp deer pellets are larger compared to those of other coexisting cervids such as hog deer or domestic animals like goats or sheep in the area, they were not considered definitive evidence on their own. Swamp deer presence was inferred in a particular area when at least two out of three indirect indicators (antlers, hoof prints, or pellets) were identified. A total of 173 presence points of swamp deer were documented from the study area through this process.

### 3.2 Environmental variables

A total of 14 variables, which included four topographic, four vegetation-related, and six anthropogenic variables, were extracted through various sources for the study (Table 1).

**Table 1:** Fourteen environmental variables including four topographic, four vegetation related and six anthropogenic variables used for Variance inflation factor (VIF) test in the study.

Source	Category	Variable	Unit
USGS	Topographic	Elevation	m
		Aspect	Degree
		Slope	Degree
GEOFABRIK		Distance to water	m
Landsat	Vegetation-related	Mean EVI, Minimum EVI, Maximum EVI (Enhanced Vegetation Index)	Dimensionless
GFC		Forest	Dimensionless
Department of Survey, Nepal	Anthropogenic	Distance to settlement	m
GEOFABRIK		Distance to the motor road	m
		Distance to path	m
		Distance to building	m
HUMDATA		Population density	Dimensionless
ICIMOD		Land use/land cover	m

### 3.3 Topographic variables

Topographic variables such as elevation, slope, aspect, and proximity to water are known to govern the distribution of large herbivores [24,83]. The digital elevation model data with a resolution of 30 m was obtained from the USGS website (https://earthexplorer.usgs.gov/). Slope and aspect were generated from the digital elevation model using ArcGIS [84]. Shapefiles with information on the sources of water were downloaded from Geobabrik (https://www.geofabrik.de/data/shapefiles.html) and converted by ArcGIS into a distance raster file. Due to the inaccessibility of high-resolution climatic variables, elevation was used as a proxy for temperature.

### 3.4 Vegetation-related variables

Vegetation-related variables are one of the most important factors influencing the distribution of herbivores like swamp deer [85,86]. Forest cover, the minimum Enhanced Vegetation Index (EVI), the mean EVI, and the maximum EVI were the four variables collected for the study. Forest cover was obtained from Ref. [87] (https://earthenginepartners.appspot.com/science-2013-global-forest).The EVI time-series data used in the study was obtained from MODIS (https://earthexplorer.usgs.gov/). A Savitzky-Golay filter was used to smooth the data in the TIMESAT program [11]. The process reduced the amount of cloud cover in the environment, which helped in visualizing the images, and then the average values were calculated for overall indices to eventually obtain the final index of EVI.

### 3.5 Anthropogenic variables

Human activities have been identified as one of the primary threats to swamp deer survival and distribution [24,82]. Anthropogenic variables such as distance to a human path, distance to roads, distance to settlements, distance to buildings, population density, and land use data were obtained as variables in this study. Data from Geofabrik's website (https://www.geofabrik.de/data/shapefiles.html) was used to extract data on the location of the path, road, and buildings. Settlements data was extracted from the Department of the Survey, Nepal, and the distance raster file was created using ArcGIS. The ICIMOD website (http://www.icimod.org/) was used to download the data on land use and land cover change. Human population density data was extracted from HUMDATA (https://data.humdata.org/).

### 3.6 Spatial thinning and reducing multicollinearity among variables

As accounting for unequal sampling bias improves model prediction efficiency [88], spatial thinning in the oversampled zone is considered effective [89]. Spatial thinning can be done by removing spatially autocorrelated points and constructing the data on location to be more calibrated and fit for evaluation [90]. We used the “spThin” package [91] in R for preprocessing the presence data to reduce the spatial autocorrelation and minimize the sampling bias. Since the final spatial resolution of our variables was 30 m, we filtered the localities and retained 158 points out of 173 presence points by removing auto-correlated points and keeping at least a 100-m distance between two presence points to reduce bias and ensure that each location is unique [92]. A variance inflation factor (VIF) test was conducted to measure the multicollinearity among variables due to its ability to explicitly measure the degree of multicollinearity between predictor variables and its sensitivity and reliability in identifying highly correlated variables [93,94]. Those variables with a high VIF (more than 10) were removed to reduce multicollinearity [95,96], hence retaining 12 out of the 14 pre-defined variables to be applied in MaxEnt modeling.

### 3.7 Prediction of the distribution of swamp deer

MaxEnt, one of the most widely used tools for modeling the distribution of species [88], was utilized to assess the potential distribution of swamp deer in this study based on the collected presence points. The sample data collected in the real-world scenario might contain bias, influencing the performance of the model and causing over-fitting. Therefore, spatial filtering was done to avoid this bias [97,98]. To make modeling robust, MaxEnt was set up to utilize 10,000 background points with a 10-fold cross-validation method to construct binary maps, with the maximum training sensitivity plus specificity as the threshold in order to determine the best model.

We used the ENMeval package in R to optimize the MaxEnt model. For this, a total of 48 possible models with different combinations of the five feature classes (FC) L, Q, H, P, and T (where L is linear, Q is quadratic, H is hinge, P is product, and T is threshold), and eight regularization multiplier (RM) values (0.5–4, 0.5) were evaluated. The ENMeval package [99] was used to test 48 parameter combinations. We used Akaike information criterion (AIC) and 5% training omission rate (OR5) alongside the difference between AUC values to identify the best-fit model [100,101]. After model optimization, the model chosen with the characteristic class (FC) = LH, RM = 0.5 and delta AIC = 0 was established as best fit model. After fixing these parameter settings, the maximum number of iterations was set to 1000, and 70% of the presence points were used to train the model, whereas the remaining data was utilized to test the model [102]. Model evaluation was done with two methods, which included threshold dependent and threshold independent methods. The value of accuracy was directly obtained from the area under the curve (AUC) in the threshold independent model. AUC values of 0.7 denote poor model performance, values greater than 0.7 denote moderate model performance, and values greater than 0.9 denote excellent model performance [103]. Whereas for the threshold-dependent model, we performed true skill statistics (TSS) in R [104]. The TSS equals sensitivity plus specificity and ranges from −1 to 1. A TSS value of less than 0 represents random performance, whereas a value close to 1 represents a perfect fit [105]. The AUC and TSS were calculated for all 10 models, and then those values were averaged to obtain the final value [83,106]. The threshold used to maximize the TSS was used to convert the continuous probability map into binary (suitable/unsuitable) map [107]. The map was classified using reclassify function of ArcGIS, and the binary classified map was intersected with the shapefiles of protected areas and district to obtain the suitable area for swamp deer.

## 4 Results

### 4.1 Distribution of swamp deer

**Fig. 2:**
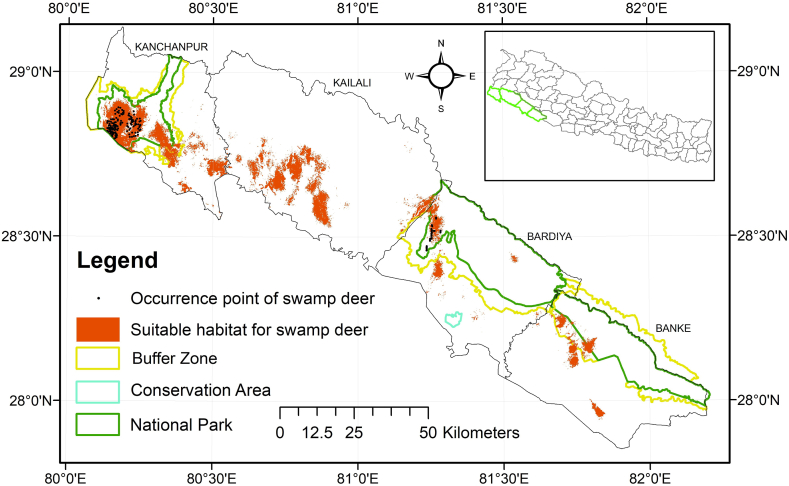
Suitable area for swamp deer (represented in red color) across four districts and three protected areas of Western Terai Arc Landscape (TAL). (For interpretation of the references to color in this figure legend, the reader is referred to the Web version of this article.)

Of the total study area, 590 km^2^ (6% of the total area) was found to be suitable for the swamp deer (Fig. 2). Among the four districts, Kanchanpur had the highest suitable habitat, followed by Kailali, Banke, and Bardia (Table 2).

**Table 2:** Suitable area (km^2^) for swamp deer within each district of Western TAL.

District	Suitable Area (km^2^)	% out of total suitable habitat
Banke	62.23933	11
Bardia	56.01832	9
Kailali	216.0011	37
Kanchanpur	256.2041	43
Total	590.4629	100

Swamp deer distribution was identified in three national parks (NPs). Shuklaphanta NP had the largest suitable habitat for the species, followed by Bardia NP and the Banke buffer zone (BZ) (Table 3). A total of 265.23 km^2^ of swamp deer habitat was inside the protected areas. Approximately 65% of the total swamp deer habitat in the study area was located outside the protected areas.

**Table 3:** Suitable area (km^2^) for swamp deer within each protected area of Western TAL.

Protected area	Area deer (km^2^)	% out of total suitable habitat
Banke Buffer Zone	24.48376	9
Banke NP	9.184139	3
Bardia Buffer Zone	3.173636	1
Bardia NP	36.55117	14
Shuklaphanta Buffer Zone	17.44644	7
Shuklaphanta NP	174.3932	66
Total	265.2323	100

### 4.2 Importance of variables to build the model

**Fig. 3:**
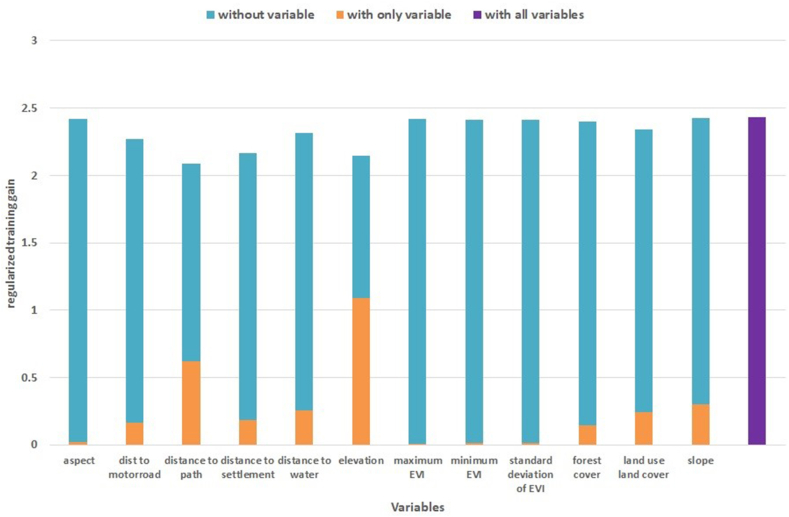
The jackknife outcomes depict the gain from regularized training for a set of 12 variables, along with their respective contribution, both without and with only a particular variable. The lighter colors are indicative of variables that exhibit a significant decrease in gain in their absence, while medium colors correspond to environmental variables that yield the highest gain when utilized in isolation. The darkest color, on the other hand, represents regularized training gain when all variables are used. (For interpretation of the references to color in this figure legend, the reader is referred to the Web version of this article.)

**Fig. 4:**
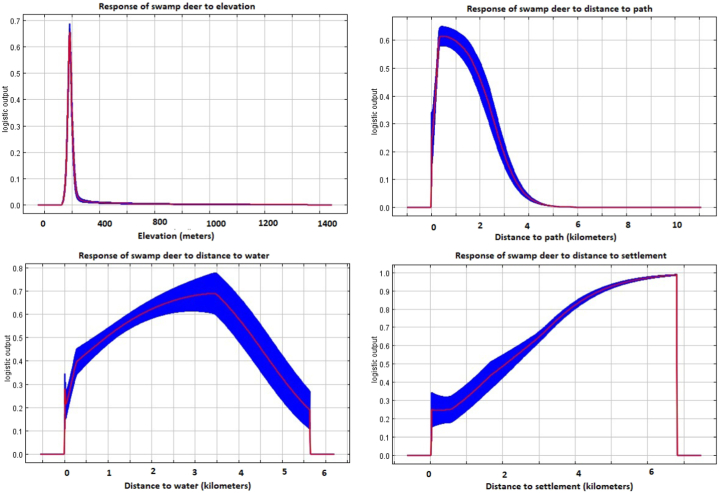
Response curves indicating the influence of four of the important variables (elevation, distance to path, distance to water, and distance to settlement) on habitat suitability of swamp deer. The X axis represents the value for four response variables, and the Y axis represent the logistic output probability. The figure represents that habitat suitability peaks at low elevation, nearby path, near water sources and far from the settlements.

Among the 12 variables used to predict the suitable habitat for swamp deer, the variables with the highest contribution to building the model were elevation, followed by distance to the path, distance to water, and distance to settlement (Fig. 3). According to the response curves, swamp deer were most likely to be found in areas with low elevation (around 200 m), near water sources (<3.5 km), far from settlements (>6 km), but close to paths (<1 km) (Fig. 4).

### 4.3 Model accuracy

The accuracy of the models generated was excellent, with an average AUC value of (0.95 ± 0.08). The TSS value generated by the models also represented a fine fit, with a value of (0.72 ± 0.06). An average threshold of 0.185 was obtained to maximize the sum of sensitivity and specificity (Table 4).

**Table 4:** The accuracy generated for the swamp deer distribution model through threshold dependent (TSS) and threshold independent (AUC) methods.

Accuracy assessment			
model	AUC	Threshold	TSS
1	0.9599	0.1853	0.7164
2	0.9685	0.176	0.802
3	0.9589	0.2074	0.6639
4	0.9569	0.1558	0.7539
5	0.9699	0.2295	0.8348
6	0.9661	0.1871	0.7997
7	0.9443	0.1744	0.6794
8	0.9447	0.1603	0.6121
9	0.9643	0.2142	0.7276
10	0.951	0.1596	0.6994
Average	0.9585	0.185	0.72892
St. dev.	0.0088	0.023749	0.065986

## 5 Discussion

Our study sheds light on important component of endemic species conservation by assessing the habitat suitability and attributes associated with the distribution of a habitat specialist mega herbivore, the swamp deer, in an important conservation landscape of Nepal. In the recent scenarios of a drastic decline in the global population of large herbivores, owing to their relatively low population densities, unique habitat requirements, and slow life history characteristics [108,109], large-scale conservation initiatives are deemed imminent for their sustainable conservation. This implies that precise information on distribution, and various habitat parameters are necessary to ensure their future survival. It was especially important to produce detailed data on the potential distribution and identify the effect of anthropogenic variables on the habitat suitability of habitat-specialist large herbivore species like swamp deer, which live in a mosaic of protected and unprotected areas close to human habitations [[110], [111], [112]]. Therefore, this study is distinct in identifying the primary distribution locations (both inside and outside protected areas), investigating the role of variables influencing the distribution, and examining the extent of protected area coverage within highly suitable areas. Thus, our findings give insight into a crucial aspect of swamp deer-centric conservation, emphasizing landscape-level population connectivity through *trans*-boundary collaboration. Besides, as there is very limited information on the distribution of the habitat specialist large herbivores across their distribution range, the findings of our study are expected to pave a path and broaden our understanding regarding the prerequisites of conserving these endemic species.

As it has been observed that using SDM approaches to construct distribution maps for habitat-specialist species such as swamp deer yields better predictions than habitat-generalist species [37,113], our study included numerous field-based presence locations and important spatial variables on a fine scale (30 m resolution), allowing us to make more accurate predictions than previous studies [114]. We did not use species absence data during our analysis because it was difficult to determine the true absence of swam deer from these mosaic environments. However, since the number of pseudo-absences has the biggest impact on model accuracy for classification and machine learning techniques, we averaged numerous runs with fewer pseudo-absences for generating the most predictive models [102]. Even though ensemble methods of multiple algorithms are known to predict with higher accuracy, given our limited resources and computational power, we utilized single algorithm modeling techniques such as MaxEnt because it is known to generate a distributional map with an accuracy comparable to ensemble techniques [115]. Nevertheless, our model predicted fairly well (AUC of 0.95 and TSS of 0.72), which is to be anticipated quite well for a habitat-specialist species [37,116]. Moreover, studies like [37], have also previously assessed the distribution and identified priority conservation areas for swamp deer through modeling and field surveys in India. However, our study stands out as the first to assess swamp deer distribution in Nepal, employing finer scale variables at 30 m resolution and focusing on the effect of anthropogenic variables on habitat suitability.

### 5.1 Influence of variables on habitat suitability

Our study's habitat suitability model identified elevation as the most important variable governing swamp deer distribution, with habitat suitability peaking around 200 m elevation. The result coincides with a previous study reporting 100–300 m as the suitable altitude range for swamp deer [12]. Elevation has been widely regarded as one of the most important predictors for species distribution, especially among mammals, all around the world [[117], [118], [119]].

We hypothesized that swamp deer would prefer habitats close to water sources and avoid areas with high anthropogenic pressure. In this context, one of the important predictors governing the swamp deer distribution in our study was the distance from water sources. Our study indicated that habitat suitability decreased as the distance from water sources increased. Swamp deer, mostly feeding on grass species and occasionally on aquatic plants [14], are generally reported to show a high preference for grassland plots with water holes [15]. The species is known to drink water at least twice a day in the winter and even more often in the summer season [17]. Even though swamp deer move 2–3 km a day [18], they are mostly found to stay within the range of water sources. Previous studies, conducted in Nepal [24] as well as India [37], have also indicated the presence of suitable areas within a few kilometers of water sources.

The habitat suitability for swamp deer increased with increasing distance from settlement, whereas the suitability decreased with increased distance from the path. Areas with high human pressure are reported to negatively influence the space use of most ungulates [120]. Since settlements are highly modified by humans to accommodate their needs, swamp deer will most likely avoid those areas. Both wild ungulates and domestic cattle are forced to share the same area for foraging in human-dominated landscapes within non-protected regions [36,121]. Since paths are usually used by humans to graze their livestock to optimum foraging grounds, it is highly likely that swamp deer will use the same paths to forage.

Similarly, a prominent importance of such anthropogenic variables (distance to settlement, distance to path, distance to water sources, etc.) in governing distribution of habitat specialist herbivore have been presented throughout several studies. These factors include effect of water availability on large herbivores [122,123], and impact of anthropogenic structure in habitat preferences [83,124], indicating the essence of managing water sources and minimizing anthropogenic encroachment for conserving these habitat specialist large herbivores all around the globe.

### 5.2 Suitable habitat for swamp deer

We hypothesized that a significant portion of the potentially suitable habitat for this species is located outside the protected areas. In this context, approximately 45% of the suitable habitat of swamp deer was found to be incorporated into three protected areas within the study site. Our study revealed that the largest suitable habitat for swamp deer existed in SNP (174.93 km^2^). At present, SNP constitutes the largest herd of swamp deer in Asia [21,29]. However, according to the SNP's records, the number of swamp deer in 2019 was 2246, down from 2301 in 2014. This decline in population could be attributed to escalating anthropogenic pressure around the national park [16], thereby limiting its population in the area. Changes in land use patterns, ineffective grassland management techniques, the spread of invasive species, and the invasion of woody perennial species within the SNP grasslands [16] are some of the anthropogenic-induced limiting factors for the expansion of the swamp deer population. The proportion of site uses by swamp deer in SNP was reported to be 0.23, which was described as being affected by natural and anthropogenic correlates [125], suggesting species-specific planning for conservation and management of swamp deer, largely focusing on grassland management. BNP, which supports 106 individuals, also harbors a significant suitable habitat for swamp deer, but the population is reported to face a continuous shortage of food, diseases, and anthropogenic stress [29,126]. As per our study, the largest continuous patch of suitable habitat lies in SNP, which connects a conservation priority area (critical corridor) identified by the study of [37]. This critical corridor connects the swamp deer population of Nepal with the Indian population within the Sharda habitat block of India, which is mostly unprotected. This critical corridor should be protected and made functional through the coordination and cooperation of Nepalese and Indian authorities. This transboundary conservation approach will help to generate and conserve the meta-population of swamp deer, which is the largest global intermixing population. Also, the Nepalese population may become genetically separated from the Indian population if this corridor is lost because of accelerating human disturbances. Besides SNP, the suitable areas of swamp deer in other protected areas (Bardia NP and Banke NP) and outside protected areas (mainly Kailali District) have fragmented patches. The largest patch of suitable habitat in the non-protected region lies within the Kailali District, which needs immediate attention for conservation because it has the potential to harbor a swamp deer population.

Since over 65% of the suitable area lies outside protected areas, this presents opportunities as well as challenges in conserving this habitat specialist species. Opportunities in the sense that the suitable area lying outside protected areas could be further expanded as suitable habitat with efficient translocations, planned conservation efforts, and grassland management endeavors. Likewise, small, isolated, and fragmented patches outside protected areas are more susceptible to human encroachment and conversion of land use, which poses great challenges to maintaining those areas intact and favorable for swamp deer. Therefore, identification of viable habitat outside protected areas and establishment of new populations through translocation could be critical for the long-term survival of species [127,128]. Thus, future conservation strategies should focus on either establishing new adjacent protected areas or extending the existing ones by including the suitable habitat patches lying outside protected areas, particularly in the far western region of Nepal, where there is an absence of connectivity and no proper networks of protected areas. This will enable the inclusion of more suitable habitats for swamp deer within the potential protected area networks. However, if the existing and future suitable habitats in protected area networks do not overlap, just expanding protected areas will aid species conservation [129,130]. This shows that appropriate corridor policies should be taken into account in the future when formulating the management plans for swamp deer within concerned protected areas, which will consequently help to strengthen the protected area networks and ultimately promote swamp deer mobility.

Alternatively, if the development or expansion of the current protected area network is not viable due to economic, political, or societal mores, then we suggest collaborative swamp deer habitat conservation initiatives with the assistance of local inhabitants within the fragmented swamp deer habitats. Also, this can be feasible in developing countries like Nepal, which is globally recognized for its successful conservation endeavors through the participatory support of the locals, for example through a community forestry program [131]. Thus, we also suggest promoting community-based conservation interventions in such fragmented habitats. This will not only benefit endemic swamp deer and other highly threatened habitat-specialist species that occur in the region but also greatly contribute to the coexistence of humans and wildlife within the landscape. Therefore, the implementation of socio-ecological arrangements for landscape-level planning in the potential suitable habitats of swamp deer and conservation activities that bind people, their socio-cultural systems, and ecological systems [[132], [133], [134], [135]] could be the appropriate course of action in this scenario.

One limitation of the study is the use of single-algorithm modeling techniques due to limited resources and computational power. Ensemble methods of multiple algorithms are known to predict with higher accuracy, and future studies could potentially utilize these methods to further improve the accuracy of predictions. Additionally, while the study focused on the effect of anthropogenic variables on habitat suitability, there may be other factors that contribute to swamp deer distribution that were not considered. Future studies could explore these additional factors, as well as incorporate data on species absences, to further improve predictive models. Furthermore, while the study focused on the distribution of swamp deer in Nepal, it did not assess the species' population size or demographic trends, which are important considerations for conservation planning. Future studies could address these gaps in knowledge to inform more effective conservation strategies.

## 6 Implications and conservation recommendations

The results of our study have important implications for the conservation of swamp deer and other habitat specialist large herbivores in the Western Terai Arc Landscape of Nepal. We identified a significant area of suitable habitat for swamp deer, but only a small portion of it is encompassed within the existing protected area system. Therefore, our study recommends expanding the protected area system to include the largest patches of suitable habitat within the non-protected regions. Additionally, connectivity between fragmented patches of suitable habitat should be increased through *trans*-boundary conservation initiatives.

To ensure sustainable conservation of swamp deer and other sympatric species, it is important to conserve water sources, manage wetlands, reduce encroachment on grasslands, and control anthropogenic expansion in the potential habitat of these species. We recommend using swamp deer as an umbrella species for habitat management, which would also benefit other habitat-specialized herbivores of grasslands and wetlands. Our study emphasizes the need for continued efforts to protect these species and their habitats through integrated conservation initiatives.

## 7 Conclusion

In conclusion, our study provides critical information on the potential suitable habitat of swamp deer and the effects of anthropogenic variables on their distribution in the Western Terai Arc Landscape of Nepal. We identified a significant area of suitable habitat for swamp deer, and our study recommends expanding the existing protected area system and increasing connectivity between fragmented patches of suitable habitat.

Our study highlights the importance of effective conservation mechanisms in mitigating the risk of extinction for habitat specialist large herbivores. We recommend using swamp deer as an umbrella species for habitat management, which would also benefit other sympatric species of grasslands and wetlands. Overall, our study emphasizes the critical role of continued efforts to protect these species and their habitats for sustainable conservation.

## Author contribution statement

Bijaya Dhami: Conceived and designed the experiments; Performed the experiments; Contributed reagents, materials, analysis tools or data; Wrote the paper.

Binaya Adhikari: Conceived and designed the experiments; Performed the experiments; Analyzed and interpreted the data; Contributed reagents, materials, analysis tools or data; Wrote the paper.

Saroj Panthi; Bijaya Neupane: Contributed reagents, materials, analysis tools or data; Wrote the paper.

## Data availability statement

Data will be made available on request.

## Additional information

No additional information is available for this paper.

## Declaration of competing interest

We declare that there are not any financial or personal relationships that have influenced this work.
